# Uncertain futures and unsolicited findings in pediatric genomic sequencing: guidelines for return of results in cases of developmental delay

**DOI:** 10.1186/s12910-023-00977-y

**Published:** 2023-11-11

**Authors:** Candice Cornelis, Wybo Dondorp, Ineke Bolt, Guido de Wert, Marieke van Summeren, Eva Brilstra, Nine Knoers, Annelien L. Bredenoord

**Affiliations:** 1 Department of Genetics, University Medical Center Utrecht, Utrecht, the Netherlands; 2 Julius Center, Department of Medical Humanities, University Medical Center Utrecht, Utrecht, the Netherlands; 3 Department of Health, Ethics & Society, Maastricht University, Maastricht, the Netherlands; 4 Department of Medical Ethics, Philosophy and History of Medicine, Erasmus Medical Center, Rotterdam, the Netherlands; 5 Department of General Pediatrics, University Medical Center Utrecht, Utrecht, the Netherlands; 6 Department of Genetics, University Medical Centre Groningen, Groningen, the Netherlands; 7 Erasmus University Rotterdam, Erasmus School of Philosophy, Rotterdam, the Netherlands

**Keywords:** Unsolicited findings, Genomic sequencing, Children, Return of results, Future autonomy

## Abstract

**Background:**

Massively parallel sequencing techniques, such as whole exome sequencing (WES) and whole genome sequencing (WGS), may reveal unsolicited findings (UFs) unrelated to the diagnostic aim. Such techniques are frequently used for diagnostic purposes in pediatric cases of developmental delay (DD). Yet policy guidelines for informed consent and return of UFs are not well equipped to address specific moral challenges that may arise in these children’s situations.

**Discussion:**

In previous empirical studies conducted by our research group, we found that it is sometimes uncertain how children with a DD will develop and whether they could come to possess capacities for autonomous decision-making in the future. Parents sometimes felt this brought them into a Catch-22 like situation when confronted with choices about UFs before undergoing WES in trio-analysis (both the parents’ and child’s DNA are sequenced). An important reason for choosing to consent to WES was to gain more insight into how their child might develop. However, to make responsible choices about receiving or declining knowledge of UFs, some idea of their child’s future development of autonomous capacities is needed. This undesirable Catch-22 situation was created by the specific policy configuration in which parents were required to make choices about UFs before being sequencing (trio-analysis). We argue that this finding is relevant for reconfiguring current policies for return of UFs for WES/WGS and propose guidelines that encompass two features. First, the informed consent process ought to be staged. Second, differing guidelines are required for withholding/disclosing a UF in cases of DD appropriate to the level of confidence there is about the child’s future developmental of autonomous capacities.

**Conclusion:**

When combined with a dynamic consent procedure, these two features of our guidelines could help overcome significant moral challenges that present themselves in the situations of children undergoing genomic sequencing for clarifying a DD.

## Background

The introduction of massively parallel sequencing techniques, such as whole exome sequencing (WES) and whole genome sequencing (WGS), has led to considerable debate about how to deal with findings that are unrelated to the initial diagnostic aim of sequencing – so-called 'unsolicited findings' (UFs) [1]. Sometimes UFs have been referred to as 'incidental findings' or 'unanticipated findings.' Such findings are different from so-called 'secondary findings' which are actively searched, yet bear no relation to the initial diagnostic aim [2].

Clinically relevant UFs may be medically actionable, i.e., there is treatment or prevention available (e.g., in the form of controls) to limit the chances of a serious or fatal outcome immediately or only in the future, or inactionable, i.e., such interventions/preventive measures are lacking. Such findings may be found in the child or the parents (when WES is conducted in trio-analysis) and may also be relevant for other family members whose DNA is not being sequenced. Additionally, clinically relevant UFs may have reproductive significance for the child, parents, and other family members. This possibility of revealing UFs can, moreover, lead to conflicts between children’s best interests and those of family/parents. This raises the question what morally responsible conditions are for (non)disclosure.

Various models and policy guidelines have been developed for dealing with such potential conflicts and tensions. Different proposals give different answers to how conflicts should be addressed between the interests of children, parents, and other relatives in choices for genetic testing and genomic sequencing as well as regarding returning results [3–11]. Policy guidelines, such as those from the American College of Medical Genetics and Genomics (ACMG) [6, 7] and the American Society of Human Genetics (ASHG) [4], have journeyed away from the broad consensus of previous debates regarding predictive testing of minors, in which testing for carrier-status and adult-onset conditions should generally be deferred until adulthood in light of future autonomy and/or welfare considerations [12–18]. The ACMG advocates actively searching for certain medically actionable adult-onset conditions, such as BRCA1 or BRCA2, but patients/parents of incompetent children undergoing sequencing are offered an opt-out from receiving these findings [6, 7]. In their 2015 policy statement the ASHG asserts that while there is no ethical requirement to actively search for secondary findings unrelated to the clinical indication for sequencing, it is ethically permissible to do so as long as there is unquestionable clinical utility for the child and/or their other family members. The ASHG also stresses that parents should be offered an opt-out from receiving secondary findings, but that the choice to opt-out may be overridden if (preventive) treatment options are available to lessen morbidity or mortality. In asserting this, onset (childhood versus adult) is not mentioned as a key factor, which opens up more room for disclosing medically actionable adult-onset conditions [4].

WES/WGS is frequently performed on children with a developmental delay (DD). In two previous empirical studies carried out by our research group we conducted semi-structured interviews with parents of children undergoing clinical WES for clarifying a DD both prior to as well as after feedback of individual results regarding their reasoning for or against wanting to receive various types of UFs and their experiences with receiving WES results. We found that it can be uncertain how these children will develop and whether they could develop capacities for autonomous decision-making in the future [19].^1^Thus, contrary to possible expectations in child cases of DD, protecting these children’s best interests may include safeguarding their future autonomy.

In this paper we show that the tendency in the policy debate to afford a weaker role to considerations regarding the child’s future autonomy is faulted, even in cases of DD. An alternative ethical understanding of children’s best interests is defended that includes protecting children’s future autonomy vis-à-vis the interests of parents/family. Based on this understanding of interests and the findings from our empirical study regarding the experiences of parents with receiving WES results for clarifying their child’s DD, new guidelines are proposed for informed consent and return of UFs. These new guidelines build on the idea of establishing a dynamic form of consent as part of standard clinical care. In dynamic consent, IT-systems are implemented that allow persons to modify their preferences for receiving results in the future [20]. Our guidelines build on the idea of ‘dynamic consent,’ but argue for limiting the scope of choices in child cases. Current guidelines from the European Society of Human Genetics from 2019 advocate that additional research “should be supported in order to help inform the development of a responsible re-contacting process and develop tools to support dynamic consent procedures.” [21].

### Qualitative findings: the undesirability of decisional Catch-22s and the desirability of default opt-ins and opt-outs

In our previous studies, we found that the specific policy configuration that parents were confronted with created decisional difficulties akin to a Catch-22, particularly when considering UFs for adult-onset conditions and carrier-status.^2^ Table 1 displays the various UF categories and policy standpoints at University Medical Center Utrecht where our research was conducted at the time of the interviews. Sometimes it was uncertain if a young child with a current DD could go on to develop the capacities needed for autonomous decision-making. A main reason for parents to consent to WES was to gain more insight into their child’s developmental potential [19]. However, center policy stipulated that parents were required to make choices regarding UFs before sequencing could be started. Some parents felt they needed at least some idea of their child’s developmental potential regarding autonomous decision-making in order to make responsible choices about UFs at present. Parents experienced this as a paradoxical situation mirroring a Catch-22.^3^

**Table 1:** UMCU’s return of UFs policy for WES in parent–child trio-analysis

Child: UF categories	Policy standpoint	Parents: UF categories	Policy standpoint
Severe conditions medically actionable^a^ in childhood	Return	Severe conditions medically actionable† in childhood	Not applicable
Severe conditions only medically actionable in adulthood	Recommend returning, but allow opt-out	Severe conditions only medically actionable in adulthood	Recommend returning, but allow opt-out
Severe medically inactionable conditions	Withhold	Severe, medically inactionable conditions	Recommend withholding, but allow opt-in
Carrier-status for severe conditions with X-linked or autosomal recessive inheritance pattern	Withhold	Carrier-status for severe conditions with X-linked or autosomal recessive inheritance pattern	Recommend withholding, but allow opt-in

^a^ ‘Medically actionable’ means that there is treatment or prevention (e.g., in the form of controls) to limit the chances of a serious or fatal outcome. For inactionable conditions such interventions/preventive measures are lacking

Another finding in our previous research was that some parents of young children never mentioned the uncertainty about their child’s autonomous developmental potential.^4^[19] These parents seemed to base their considerations favoring acceptance/decline of UFs on the assumption that their child would become autonomous, even though this was questionable due to their child’s current DD and/or co-occurring health problems. A failure to recognize the uncertainty about the child's developmental potential may also undermine the capacity of responsible decision-making by parents. These intricacies should be accounted for when developing informed consent and policy for WES.

An additional finding of our previous study was that the utilization of defaults (‘disclose, but allow opt-out,’ ‘withhold, but allow an opt-in’) for various categories of UFs was seen as desirable by parents.^5^ Parents felt that a policy that offered no choices over what UFs to hear would not be able to do justice to the context-specific factors of their situations. Moreover, having choices was valued in virtue of its importance for well-informed decision-making, since it prompts critical reflection on the potential negative and positive consequences of receiving or declining certain types of UFs for one’s own unique situation. Supplementing choice with defaults (see Table 1) was viewed as positive. This was because, as parents explained, professionals most likely had good reasons for advising to disclose/withhold certain information and this prompted parents to come up with new types of considerations for accepting/declining that information.

### Parental autonomy and the best interests of the child

Autonomous action, for our purposes, is self-governed action. Such actions are intentional, free, and unforced. Persons should be able to make their own decisions insofar as other persons’ rights are not violated.

As caretakers, parents should also enjoy considerable decisional space to make choices on their child’s behalf; parental autonomy usually deserves respect. However, parents also have duties toward their child, derived from what is in their child’s best interest, that put limits on how they may exercise their parental autonomy. If a child can be legitimately expected to develop autonomous capacities, parents have the duty to ensure conditions for developing and executing these capacities. Parents should, thus, foster their child’s (developing) autonomy. Various ethical theories support a high appreciation for respecting persons’ autonomy and, consequently, for safeguarding children’s future autonomy. Importantly, protecting future autonomy requires refraining from deciding on central aspects of children’s lives that could in principle be postponed until the child can decide for itself [22]. This includes certain decisions about gaining information about their genetic make-up. Preserving future autonomy is in a child’s best interest if they can become autonomous.

Often children with a current DD, however, will not go on to develop autonomous capacities. But, as findings from our empirical study indicate, this can be uncertain to differing degrees. The epistemological possibilities should be viewed as being situated on a continuum. One extreme is characterized by an abundance of evidence that the child is unable to develop autonomous capacities (high confidence) and on the other extreme are situations characterized by a lack of evidence for whether the child can develop autonomous capacities (low confidence). For children for whom we have high confidence that they are unable to develop autonomous capacities, future autonomy cannot be the justification for setting limits to parental decision-making regarding return of UFs. For these children, acting in their best interests requires acting in accordance with their current and future welfare.

In cases where the evidence is inconclusive or wholly lacking regarding the child’s inability to develop such capacities, it is morally required to operate on the assumption that there is a possibility that the child could develop autonomous capacities. Thus, the less we can confidently assert that a child is unable to develop autonomous capacities, the more we must acknowledge future autonomy considerations in our argumentation of what constitutes a child’s best interests. Subsequently, as argued above, acknowledging future autonomy considerations sets limits to what UFs may be returned/withheld.

In medical practice, children’s best interests and respecting parents’ autonomy impose moral duties on healthcare professionals. Professionals have a duty to respect medical decisions that parents make for their child, since parents are usually in the best position to know what is in their child’s best interests. Nevertheless, this duty to respect parents’ decisions is constrained by a duty to act in the best interests of children as providers of medical care. If a professional has strong grounds for thinking that a parental choice is counter to the child’s best interests, they must protect the child they are treating.

## Discussion

### Guidelines for informed consent & return of results

**Fig.1:**
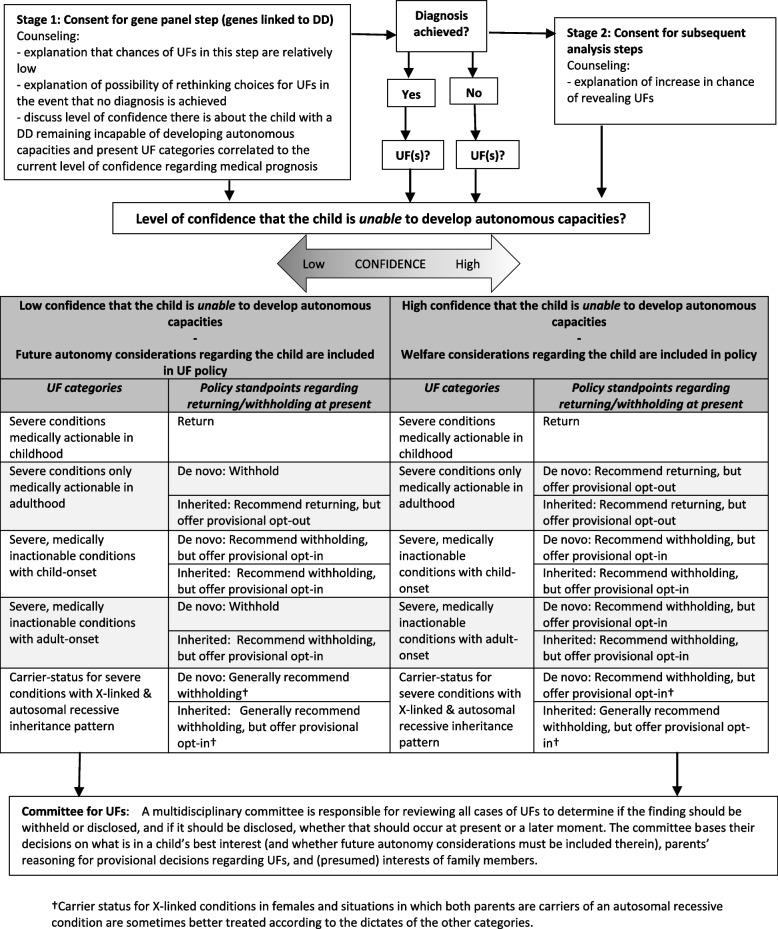
WES (trio-analysis) in pediatric cases of DD: consent and return of UFs guidelines

To tailor informed consent and return of UFs to the situations of children undergoing WES in trio-analysis (in which the parents’ and child’s DNA is sequenced and interpreted) for clarifying a DD, the guidelines we propose contain two important features. Firstly, instead of viewing the obtainment of informed consent as a snapshot moment, a staged approach should be adhered to. The main benefit of this approach is that it can limit the decisional impasses resembling Catch-22s experienced by parents whose children’s future autonomy cannot be confidently estimated. Secondly, guidelines for withholding/returning of UFs (at present) differ and correlate with whether a child’s case is one in which we have higher or lower confidence regarding the child’s inability to develop autonomous capacities. Due to the potential uncertainty associated with predictions regarding the child’s development of autonomous capacities, the complexity and changing nature of genetic information, and potential competing interests, we argue that for some UF-categories, parents should be offered what we refer to below as ‘provisional choices,’ supplemented by default policy options (opt-ins/opt-outs) in order to support well-informed decision-making. The choices are provisional, because they are subject to being reviewed, and potentially overruled, by a multidisciplinary committee. The committee bases their decisions on the following factors. First, what is in a child’s best interest and whether future autonomy considerations must be included therein. Second, what parents’ reasoning for their provisional decisions regarding UFs was at the time of consent should they choose in opposition to the default (i.e., opting in when it is recommended to withhold the information or opting-out when it is recommended to disclose the information). Third, what the (presumed) interests of other family members are (whose DNA has not been sequenced) in the information revealed by WES. Figure 1 displays the guidelines. Below we explain these features in greater detail.

### Staging informed consent

The first feature of our guidelines draws on the idea that informed consent for WES should be split up into stages according to steps in trio-analysis. Staging the consent process in this way limits the Catch-22 situation parents will otherwise find themselves in. An added advantage of staged consent is that it views consent as a time-intensive process, not as a snapshot moment: persons need time to weigh alternatives and decide between them, and staged consent provides this [23, 24].

Steps in trio-analysis of WES-data for clarifying a DD at UMCU are as follows. First, a targeted gene panel is used to analyze ± 1000 genes associated with DD. If this does not result in a diagnosis, the second step is to analyze all genetic variants that a child has but the parents lack, so-called ‘de novo’ variants and all inherited variants for which the child is homozygous, compound heterozygous, or which are located on the X-chromosome. These steps constitute ‘filters’ intended to efficiently achieve a diagnosis, while avoiding UFs as much as possible, which is strongly advised by European guidelines [11]. The chance of UFs increases from the first to second step. At the time of the interviews at UMCU parents were required to consent to all analysis steps and any UFs that may be found before sequencing could begin.

By contrast, in our staged approach, consent is first obtained for the gene panel step and, if no diagnosis is attained, then consent is obtained for the analysis of de novo variants and the mentioned inherited mutations. The increase in chance of revealing UFs should be explained to parents during counseling and they should be directed to make conscious decisions about UFs in the unlikely event that one should be discovered in the gene panel step. Parents may rethink their choices regarding UFs as they move to the next phase and be told that they may withdraw their consent for WES after stage 1.

The diagnostic yield from WES in the first analysis step is roughly 25 – 50%; [25] thus, for a number of children a diagnosis can be reached in the first step. This means that parents could at least consent to the first step of WES and still have the chance of receiving a diagnosis, but with the least possible risk of receiving UFs. Hence, our approach eliminates a portion of the decisional deadlock parents find themselves in, thereby facilitating access to their children’s care. Moreover, even if parents are not in such a Catch-22 situation, staging the consent process can still be beneficial to well-informed decision-making because it allows persons to rethink choices for UFs and because it structures decision-making according to risk–benefit trade-offs, i.e., the benefits of obtaining a diagnosis (swiftly) versus the possible negative effects posed by revealing an UF [26].

### Return of UFs appropriate to level of confidence regarding the child’s development of autonomous capacities

A second feature of our guidelines is that disclosing or withholding a certain type of UF is connected to the level of confidence there is that a child with a DD is unable to develop autonomous capacities. In asserting this, clinicians are being asked to make a prediction about whether the child could go on to develop autonomous capacities needed for making decisions about UFs. Such predictions may be based on, but not be limited to, such factors as: the current health conditions the child is experiencing that a diagnosis through WES may help to clarify, the prognoses attached to those conditions, the child’s developmental progress to date and their age.

For cases in which there is low or no confidence about the child’s inability to develop autonomous capacities and thus in which it cannot be ruled out that the child could develop such capacities, a cautious approach to returning UFs for carrier-status, conditions only medically actionable in adulthood, and adult-onset conditions lacking medical actionability ought to be taken. When there is high confidence about the child’s inability to develop autonomous capacities, i.e., there is abundant evidence that she/he cannot develop autonomous capacities, then protecting her/his best interests should be understood in terms of welfare; more decisional discretion is, thus, afforded to parents.

Affording parents decisional discretion, as explained above, is morally required out of respect for their autonomy. As parents explained in our study, a one-size-fits-all policy is unable to accommodate the diversity of persons’ situations in that respect and having at least some choices is conducive to informed consent. Moreover, our empirical results indicate: offering choices strongly encourages critical reflection on the possible consequences (e.g., regarding insurance coverage, emotional impact) of receiving/not receiving certain genetic knowledge, thereby aiding in weighing alternatives, which is an important constituent of informed consent.^6^

Where parents are allowed choices over UFs, our guidelines stipulate offering default policy options (‘recommend returning, but offer opt-out’ and ‘recommend withholding, but offer opt-in’) that should be used to support parents’ well-informed decision-making. Our empirical findings indicate that presenting policy for UFs in this manner can help parents fathom new kinds of considerations favoring withholding/returning UFs, which is conducive to informed consent.^7^

A further complexity inherent to trio-analysis that can potentially give rise to conflicts of interest between what parents might want to know and what is in their child’s best interest (whether according to the future autonomy interpretation or the welfare interpretation), is that it is impossible to disclose an inherited UF in the case of the parent(s), while withholding the finding concerning the child. This is due to how sequencing data is analyzed: if a UF is found in the parents this will always be because there was a UF found in the child. Our guidelines, thus, make a distinction between inherited UFs and de novo UFs found in the child. In cases where we have low confidence that a child with a current DD is unable to develop autonomous capacities, parents are only afforded a limited amount of discretion over de novo UFs in order to safeguard the child’s autonomy.

Furthermore, the choices parents make about UFs under our guidelines are ‘provisional’ choices, since it may sometimes be morally required to overrule the choice if competing interests are at stake. In agreement with Holm et al., due to the complexity of genetic information, we recommend that a multidisciplinary committee should review all cases of UFs and determine whether parents’ provisional choices should be upheld/overruled [8].

In cases where the committee decides that a UF should be withheld at present, clear procedures regarding information storage and re-contact need to be in place for possible future disclosure. The committee is responsible for reviewing and advising on disclosure plans. We recognize that there are presently certain legal and practical challenges involved in storing UFs for possible future disclosure and that an adequate IT system is currently lacking. The guidelines developed here implicitly emphasize the moral duties we have to investigate the (possible) realization of these systems. In that sense, our guidelines are based on the ideal rather than on the current state of affairs. Several initiatives are being undertaken in bio-banking research that employ a dynamic approach to consent that use IT-systems to allow persons to modify their preferences for receiving results in the future [20]. Our guidelines build on the idea of ‘dynamic consent,’ but argue for limiting the scope of choices in child cases.

### Low confidence that the child is unable to develop autonomous decision-making capacities & return of UFs

Below, we explain the disclosure directives for cases in which there is low confidence that the child is unable to develop the capacities necessary for autonomous decision-making. In such cases, it is advised to take measures that help protect the child’s possibilities for future autonomous decision-making:

- *Severe conditions medically actionable in childhood*. It is always in a children’s best interests to disclose these types of UFs. Life is a prerequisite for an autonomous future and good childhood health is conducive to autonomy. This standpoint is consistent with current policy guidelines/models in pediatrics [3–11]. Examples of such conditions would include child-onset forms of cancer or heart disease.
- *Severe conditions only medically actionable in adulthood. *A BRCA1 or BRCA2 mutation are examples. For these types of conditions, the requirements for disclosing or withholding depend on whether the mutation is inherited or de novo. If de novo, the information should be saved for possible future disclosure, since it is unclear whether the child could develop autonomous capacities and could decide for themselves at a later point in time. Alternatively, if it becomes clearer that the child will always remain under the care of their parents, then parents may be given this information at that point in time. If the mutation is inherited, however, then it constitutes information that could be relevant for the parents’ and relatives’ health and that is medically actionable. However, delayed disclosure may sometimes be appropriate in such situations, e.g., mortgage ineligibility. Next to such possibilities, the committee’s deliberations should also include the child’s interest in growing up with healthy parents and risks to family who could not otherwise have known this information.
- *Severe, medically inactionable conditions with child-onset. *For medically inactionable child-onset conditions, parents may provisionally opt to receive this information for both de novo and inherited mutations. Especially in cases where a child-onset condition is likely to cause severe cognitive impairment/fatality in childhood, future autonomy considerations cannot offer the justification for withholding this information. The grounds for returning such UFs then shift to the welfare understanding of best interests. Moreover, disclosing these types of findings could help families avoid yet another diagnostic odyssey, and inherited UFs could have major implications for parents’/relatives’ reproductive decision-making.
- *Severe, medically inactionable conditions with adult-onset. *UFs regarding adult-onset, severe medically inactionable conditions, if de novo, should generally not be disclosed at present in order to safeguard the child’s possibilities for future autonomous decision-making. Inherited variants in this category should generally not be disclosed for the same reason, but exceptions may apply if there are major implications for parents’/adult family members’ reproductive decision-making (i.e., if there is a high probability that a future child may be affected). Therefore, parents may provisionally opt to receive this information. Examples in this context would include adult-onset neurodegenerative disorders such as ALS.
- *Carrier-status for severe conditions with X-linked & autosomal recessive inheritance.*

UFs for carrier-status regarding severe conditions with X-linked and autosomal recessive inheritance should be grouped into a separate category in order to emphasize to parents that such findings largely derive their possible significance for persons from the potential consequences for reproductive decision-making. For most female carriers of X-linked conditions, the risks posed will never materialize, and even if they do, the symptoms will usually be milder than those of affected males (depending on the risk involved for an affected female the committee may sometimes be justified in treating the UF as falling under one of the other UF categories discussed above). However, all (future) sons of these carriers have a 50% chance of being affected. Parents may provisionally opt to receive inherited UFs for female carrier-status for X-linked conditions for reproductive reasons, but we advocate a cautious approach toward disclosing de novo variants in cases where there is low confidence in the child’s inability to develop autonomous capacities. In contrast to X-linked carrier-status, UFs for autosomal recessive carrier-status do not pose risks to carriers, and can only have major implications for reproductive decision-making if both parents are carriers of that condition. Due to the manner in which sequencing data is analyzed, this can only be revealed if a UF is found in the child that predisposes him/her to developing the condition in question. If the child is predisposed to developing a severe condition as a result of both parents’ autosomal recessive carrier-status, then the decision for disclosing/withholding should occur according to the dictates of the requisite category. Our guidelines allow parents to provisionally opt to receive inherited UFs that pose major reproductive implications. This generally precludes disclosing UFs for autosomal recessive carrier-status if only the child and one parent are carriers. Yet when UFs can be said to pose major reproductive implications, we recognize that some parents may still wish to do without knowledge of their own carrier-status, even if they do wish to (or will, as outlined above) receive UFs for the predisposition in the child (which ought to be handled according to the requisites of the respective categories discussed above). This may be due to the fact that parents may not find such information relevant to their reproductive decision-making. In its deliberations regarding withholding/disclosing such UFs, the committee must also consider whether other children of the parents could be predisposed to developing a severe, medically actionable condition, which could warrant overruling the couple’s preference not to receive this information.

### High confidence that the child is unable to develop autonomous decision-making capacities & return of UFs

For some children with a DD, abundant evidence may already be available that they are unable to develop autonomous capacities and we can be said to have higher confidence that this will remain the case. In these cases, more provisional discretion should be offered to parents over a broader range of UF-categories under the condition that this is in accordance with the child’s welfare. UFs for severe conditions medically actionable in childhood should, however, always be disclosed to protect the child’s immediate health interests.

For UFs related to severe conditions only medically actionable in adulthood, it is generally warranted to disclose such results both for the child and the parents. Just as we argued in cases of low confidence, all children have an interest in having healthy parents. However, we also recognize that some parents, especially those of younger children, may wish to delay gaining knowledge of de novo UFs until closer to the moment at which medical actionability becomes available. Our guidelines allow parents to provisionally opt-out of receiving both de novo and inherited variants at present as long as clear plans are in place for future disclosure of the UF pertaining to the child. If parents wish to delay disclosure, the committee must also consider whether there could be any risk to other family members.

For both de novo and inherited UFs pertaining to severe medically inactionable conditions with child- or adult-onset, and those for carrier-status related to X-linked and autosomal recessive conditions, our guidelines grant parents much more provisional discretion to receive this information at present based on nonmedical utility considerations, and in the case of inherited variants based on reproductive considerations, as long as this accords with the child’s welfare.

## Conclusion

A novel feature of our guidelines, that to date has not appeared in other models or policies regarding informed consent and return of results for massively parallel sequencing technologies such as WES and WGS, is that it stipulates different disclosure requirements that correlate to the degree of confidence there is about the child’s inability to develop autonomous capacities related to decision-making. In doing so, the uncertainty that can exist about development of such capacities especially in cases of young children with a DD is acknowledged. In comprising the guidelines, we have also drawn from the work of other researchers in which the consent process is staged and in which defaults are utilized in the form of provisional opt-ins/opt-outs to facilitate parents’ well-informed decision-making [24, 27]. Although using defaults is common practice in genetics, results from our empirical studies highlight what the relevance is for assisting parents in making their own well-informed decisions. All of the mentioned elements in these guidelines contribute to overcoming significant moral challenges that present themselves in the situations of children undergoing genomic sequencing in trio-analysis for clarifying DD. Future research should focus on solving implementation challenges pertaining to information storage, re-contact procedures, and formalizing committees’ deliberation processes. It should also be explored whether a staged approach to consent for WES is appropriate in all contexts in which WES is used on minors. This could include further examination of contexts in which achieving a rapid diagnosis is essential, since a two-step staged approach does lengthen the term of receiving results. For example, such as in the case of a neonate with a severe condition admitted to the neonatal intensive care unit. Further development of the guidelines must be seen as an iterative process: the guidelines should be the subject of continual evaluation and updated as new developments and academic insights emerge.

## Data Availability

The qualitative empirical data that support the findings of this study are available on request from M.J.H. van Summeren. The data are not publicly available due to legal restrictions.

## Abbreviations

WES: Whole exome sequencing
WGS: Whole genome sequencing
UF(s): Unsolicited finding(s)
DD: Developmental delay
ACMG: American College of Medical Genetics and Genomics
ASHG: American Society of Human Genetics

## Footnotes

1 Cornelis, C., Tibben, A. Brilstra, E., Bolt, I., van Summeren, M., Knoers, N., Bredenoord, A. (2023). [Unpublished data, manuscript under consideration at Mol. Genet. Genomic Med.].

2 Cornelis, C., Tibben, A. Brilstra, E., Bolt, I., van Summeren, M., Knoers, N., Bredenoord, A. (2023). [Unpublished data, manuscript under consideration at Mol. Genet. Genomic Med.].

3 Cornelis, C., Tibben, A. Brilstra, E., Bolt, I., van Summeren, M., Knoers, N., Bredenoord, A. (2023). [Unpublished data, manuscript under consideration at Mol. Genet. Genomic Med.].

4 Cornelis, C., Tibben, A. Brilstra, E., Bolt, I., van Summeren, M., Knoers, N., Bredenoord, A. (2023). [Unpublished data, manuscript under consideration at Mol. Genet. Genomic Med.].

5 Cornelis, C., Tibben, A. Brilstra, E., Bolt, I., van Summeren, M., Knoers, N., Bredenoord, A. (2023). [Unpublished data, manuscript under consideration at Mol. Genet. Genomic Med.].

6 Cornelis, C., Tibben, A. Brilstra, E., Bolt, I., van Summeren, M., Knoers, N., Bredenoord, A. (2023). [Unpublished data, manuscript under consideration at Mol. Genet. Genomic Med.].

7 Cornelis, C., Tibben, A. Brilstra, E., Bolt, I., van Summeren, M., Knoers, N., Bredenoord, A. (2023). [Unpublished data, manuscript under consideration at Mol. Genet. Genomic Med.].
